# Clusters of preterm live births and respiratory distress syndrome-associated neonatal deaths: spatial distribution and cooccurrence patterns

**DOI:** 10.1186/s12889-022-13629-4

**Published:** 2022-06-20

**Authors:** Ana Sílvia Scavacini Marinonio, Daniela Testoni Costa-Nobre, Milton Harumi Miyoshi, Rita de Cassia Xavier Balda, Kelsy Catherina Nema Areco, Tulio Konstantyner, Mandira Daripa Kawakami, Adriana Sanudo, Paulo Bandiera-Paiva, Rosa Maria Vieira de Freitas, Lilian Cristina Correia Morais, Mônica La Porte Teixeira, Bernadette Cunha Waldvogel, Maria Fernanda Branco de Almeida, Ruth Guinsburg, Carlos Roberto Veiga Kiffer

**Affiliations:** 1 Departamento de Pediatria, Escola Paulista de Medicina – Universidade Federal de São Paulo (UNIFESP), Rua Marselhesa 630, São Paulo, Vila Clementino 04020-060 Brazil; 2 Fundação Sistema Estadual de Análise de Dados (SEADE Foundation), Avenida Professor Lineu Prestes, 913 - Cidade Universitária, São Paulo, 05508-000 Brazil

**Keywords:** Infant, Premature, Respiratory Distress Syndrome, Newborn, Neonatal Mortality, Epidemiological Studies, Spatial Distribution, Population

## Abstract

**Background:**

Prematurity and respiratory distress syndrome (RDS) are strongly associated. RDS continues to be an important contributor to neonatal mortality in low- and middle-income countries. This study aimed to identify clusters of preterm live births and RDS-associated neonatal deaths, and their cooccurrence pattern in São Paulo State, Brazil, between 2004 and 2015.

**Methods:**

Population-based study of all live births with gestational age ≥ 22 weeks, birthweight ≥ 400 g, without congenital anomalies from mothers living in São Paulo State, Brazil, during 2004–2015. RDS-associated neonatal mortality was defined as deaths < 28 days with ICD-10 codes P22.0 or P28.0. RDS-associated neonatal mortality and preterm live births rates per municipality were submitted to first- and second-order spatial analysis before and after smoothing using local Bayes estimates. Spearman test was applied to identify the correlation pattern between both rates.

**Results:**

Six hundred forty-five thousand two hundred seventy-six preterm live births and 11,078 RDS-associated neonatal deaths in São Paulo State, Brazil, during the study period were analyzed. After smoothing, a non-random spatial distribution of preterm live births rate (*I* = 0.78; *p* = 0.001) and RDS-associated neonatal mortality rate (*I* = 0.73; *p* = 0.001) was identified. LISA maps confirmed clusters for both, with a negative correlation (*r* = -0.24; *p* = 0.0000). Clusters of high RDS-associated neonatal mortality rates overlapping with clusters of low preterm live births rates were detected.

**Conclusions:**

Asymmetric cluster distribution of preterm live births and RDS-associated neonatal deaths may be helpful to indicate areas for perinatal healthcare improvement.

**Supplementary Information:**

The online version contains supplementary material available at 10.1186/s12889-022-13629-4.

## Background

Approximately 15 million preterm births occur per year worldwide, representing 11% of the total yearly births. Around 1 million deaths per year occur in infants born with gestational age < 37 weeks [1], and respiratory distress syndrome (RDS) is one of the most frequent causes of death in this group [2]. In the United States, RDS prevalence was 361 per thousand live births with gestational age less than 34 weeks, in 2014 [3]. This incidence is inversely proportional to gestational age, occurring in 86% of infants with 28 weeks and in 98% of those with 24 weeks [4].

Over the years, higher survival rates of preterm infants with RDS have been observed in high income countries, driven by management advances [3–5], including the reduction of RDS frequency and/or its severity by peripartum care with antenatal corticosteroids, and minimization of the disease consequences with surfactant administration and respiratory support strategies, all highly related to the quality of neonatal care [5, 6]. Despite these advances, RDS management practices vary among regions influenced by health care accessibility, local or regional health policies, and health infrastructure [7, 8]. In this context, RDS is still associated with unfavorable outcome in very low birth weight infants [9], and it is considered an independent risk factor for neonatal death among preterm infants in low- and middle-income countries (LMIC) [10].

In Brazil, an upper middle-income country [11], RDS rate among infants with 24–34 weeks of gestational age delivered in tertiary centers located in the South, Southeast, and Northeast regions of the country, was 49.8% in 2011–2013 [12]. Considering the period of 2011–2017 in a single center of a Southern Brazilian State, RDS occurred in 4.2% of preterm infants with 36 weeks of gestational age, and in 100% of those with 22 weeks [13]. In centers that participated in the Brazilian Surfactant Collaborative Group, RDS-mortality among preterm infants was 33 to 40% in 2005/2006, reaching 14 to 21% for those with birthweight between 1000 and 1499 g, and 68% of those < 750 g [14].

São Paulo State is the richest, most populous, and urbanized subnational Brazilian entity, with good socioeconomic indicators, but important social and economic inequities [15]. In 2010, the average per capita income among São Paulo State municipalities varied almost 18 times and 16.5% of the population lived in a situation of high social vulnerability, with discrepancies in education, housing conditions, access to primary health care [16, 17], and qualified perinatal care, which have important impact on neonatal mortality [18]. In 2006–2010, the neonatal mortality rate among São Paulo State municipalities varied between 6.9 and 16.9 per thousand live births [19], however, the study of cause specific neonatal deaths, particularly of RDS-associated neonatal deaths, is scarce for the state and its municipalities.

Considering RDS incidence and mortality among preterm infants [13, 14], and the neonatal mortality variation across the state [19], it is possible that the distribution of RDS-associated neonatal deaths in preterm live births also varies among São Paulo State municipalities. Identification of these variations in RDS-associated neonatal mortality throughout the state may help to design public health policies for the prevention and management of the disease, contributing to decrease the burden of prematurity as a cause of neonatal deaths across the State.

Given this scenario, this study aimed to identify clusters of preterm live births and RDS-associated neonatal deaths and their cooccurrence pattern in São Paulo State, Brazil, between 2004 and 2015.

## Methods

**Fig. 1:**
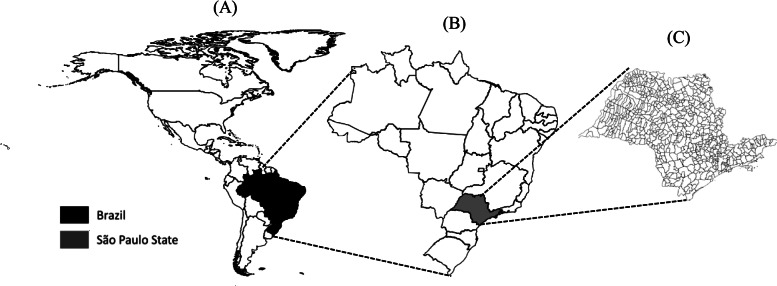
Location of São Paulo State and its municipalities in Brazil, and America. **A** American Continent; **B** Brazil; **C** São Paulo State and its municipalities

This is a population-based study of live births with gestational age ≥ 22 weeks, birthweight ≥ 400 g, and without congenital anomalies registered in the birth or in the death certificate, from mothers living in São Paulo State, Brazil, between 2004 and 2015. São Paulo State has 645 substate divisions (or municipalities) that contain 41,262,199 inhabitants, which represents more than a half of the total population living in the Southeast region of Brazil [15]. São Paulo State and its municipalities’ location in Brazil and America are showed in Fig. 1. The American continent map and geographic coordinates and spatial files of São Paulo State and the respective municipalities in shape format (.shp) were obtained from the “Instituto Brasileiro de Geografia e Estatística” (IBGE) [20].

The study was approved by the Ethics Committee of Universidade Federal de São Paulo, under the number 4.055.489, with informed consent waive (unidentified database).

The data were extracted from two databases of Fundação Sistema Estadual de Análise de Dados (SEADE Foundation), one containing the deaths certificates and the other the live births certificates information covering 99.8% and 99.5% of all occurrences in the State, respectively [21]. SEADE Foundation made the deterministic linkage of death and live birth records to identify the birth information for all infants who died within 365 days after birth [22]. To adapt the data set to the study design, a final database was created by integrating the birth variables common among the neonatal death records, retrieved from the linked database, with the live birth records [23].

The following definitions were used: preterm live births: live births with gestational age < 37 weeks; neonatal death: any death occurring between 0 and 27 days after birth; RDS-associated neonatal death: any neonatal death with the following ICD 10 codes in any line of the Death Certificate: P22.0 (respiratory distress syndrome of newborn) or P28.0 (primary atelectasis of newborn) [24]; preterm live birth rate: number of preterm live births per thousand live births; and RDS-associated neonatal mortality rate: number of RDS-associated neonatal deaths per thousand preterm live births.

The demographic characteristics considered for preterm live births and RDS-associated neonatal deaths from 2004 to 2015 were: gestational age (stratified as 22^0/7^–27^6/7^, 28^0/7^–31^6/7^ and 32^0/7^–36^6/7^ weeks), birthweight (< 1500 g and ≥ 1500 g), sex (male or female), 5th minute Apgar score (< 7 and ≥ 7), maternal age (< 20, 20–34 and ≥ 35 years), maternal schooling (≤ 7 or > 7 years of study), parity (primiparous and multiparous), number of prenatal care visits (< 4 and ≥ 4), pregnancy type (single or multiple), delivery mode (vaginal or cesarean section), and place of delivery (hospital or other). For RDS-associated neonatal deaths, age of death was evaluated. Municipality of maternal residence, municipality of infants’ birth and neonatal deaths were analyzed, considering the 645 municipalities of São Paulo State.

Initially, crude rates of preterm live births and RDS-associated neonatal mortality per municipality were mapped. Preterm live births crude rates were calculated by dividing the number of preterm live births by the total number of live births included (by municipality). For RDS-associated neonatal mortality rates, calculation was based on the total RDS-associated neonatal deaths divided by total preterm live births (by municipality). Then, to evaluate spatial autocorrelation presence, maps were generated and hierarchically analyzed by first- and second-order effects. First-order effects were visually explored for clusters by spatial moving average [25] of preterm live births and RDS-associated neonatal mortality rates per municipality. Following, the same rates were explored for second-order effects by Global Moran Indicators (I), that measures spatial autocorrelation among all municipalities in study, and tests the null hypothesis of random distribution of observed values. Results are shown in the range of -1 to + 1 and considered significant if *p* < 0.05. Significance was analyzed using 999 random permutations by Monte Carlo permutation. Null results (I = 0; *p* < 0.05) suggest random distribution; positive values (I > 0; *p* < 0.05) suggest non-random distribution with positive spatial autocorrelation (similarity among municipalities rates); and negative results (I < 0; *p* < 0.05) suggest non-random distribution with negative spatial autocorrelation (dissimilarity among municipalities rates). If first- or second-order effects suggested the presence of spatial autocorrelation, a smoothing technique with local empiric Bayesian estimator was applied in order to reduce the impact of outlier areas [25, 26]. Then, Global Moran Indicators were applied after a smoothing procedure to test the spatial autocorrelations of the adjusted rates. Finally, if spatial autocorrelation was found, Local Indicators of Spatial Association (LISA) cluster maps were generated to locate clusters of municipalities with similar values. LISA was analyzed using 9,999 random permutations by Monte Carlo permutation for assessing the significance of rate correlation. Clusters of preterm live births and RDS-associated neonatal mortality in the São Paulo State were then considered if LISA cluster maps showed correlation of rates between municipalities greater than 95% [27]. All spatial analysis tools are included in the software package (TerraView® version 5.5.0, Instituto Nacional de Pesquisas Espaciais, São José dos Campos, Brazil).

Finally, Spearman test was applied to explore the correlation between preterm live births and RDS-associated neonatal mortality rates for all municipalities, and a map overlapping the clusters of both rates (i.e., preterm live births rate and RDS-associated neonatal mortality rate) identified in each specific LISA cluster map after smoothing was generated.

All analytical procedures were performed using Stata 15.1® (StataCorp LLC, Texas, USA) and TerraView® software version 5.5.0 (Instituto Nacional de Pesquisas Espaciais, São José dos Campos, Brazil).

## Results

**Fig. 2:**
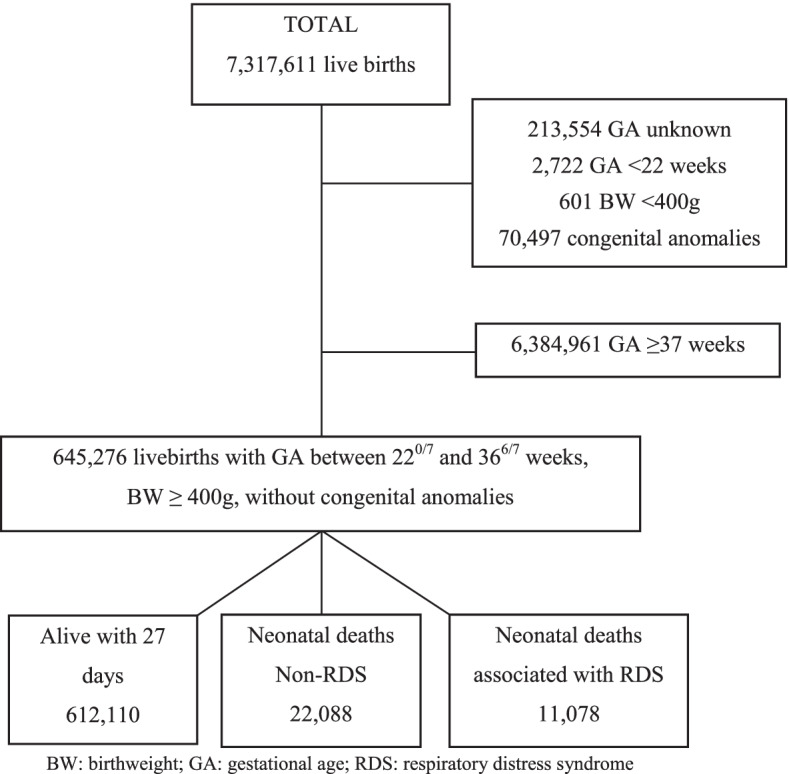
Flowchart of the studied population. *BW* Birthweight, *GA* Gestational age, *RDS* Respiratory distress syndrome

From 2004 to 2015, there were 7,317,611 live births in São Paulo State, Brazil (Fig. 2). Among 7,030,237 live births ≥ 22 weeks of gestational age, ≥ 400 g, and without congenital anomalies, 645,276 (9.2%) were preterm live births.

Most neonatal and maternal demographic variables were almost fully available, with the exception of maternal schooling, parity, prenatal care visits, and 5^th^ minute Apgar score (Table 1). Among all preterm live births, 640,955 (99.3%) were delivered in hospitals. Among infants that died with RDS, the death occurred at a mean time of 48 (P25-75: 16–120) hours after birth.

**Table 1:** Preterm live births and RDS-associated neonatal deaths demographic characteristics, São Paulo state, Brazil, 2004–2015

	Preterm live births n (%)	RDS-associated neonatal deaths n (%)
*Maternal age (years)*	*n* = 645,253	*n* = 11,078
< 20	109,089 (16.9)	2,504 (22.6)
20–34	433,067 (67.1)	7,161 (64.6)
≥ 35	103,097 (16.0)	1,413 (12.8)
*Maternal schooling (years)*	*n* = 504,995	*n* = 9,305
≤ 7	126,382 (25.0)	2,850 (30.6)
*Primiparous*	*n* = 487,941	*n* = 9,485
Yes	213,787 (43.8)	4,276 (45.1)
*Prenatal care visits*	*n* = 633,801	*n* = 10,693
≥ 4	556,029 (87.7)	6,793 (63.5)
*Multiple pregnancy*	*n* = 645,110	*n* = 11,078
No	556,993 (86.3)	9,209 (83.1)
*Delivery*	*n* = 644,905	*n* = 11,075
C-section	388,302 (60.2)	4,707 (42.5)
*Gestational age*	*n* = 645,276	*n* = 11,078
22^0/7^–27^6/7^ weeks	32,987 (5.1)	6,779 (61.2)
28^0/7^–31^6/7^ weeks	63,106 (9.8)	3,032 (27.4)
32^0/7^–36^6/7^ weeks	549,183 (85.1)	1,267 (11.4)
*Sex*	*n* = 645,276	*n* = 11,078
Male	340,101 (52.7)	6,336 (57.2)
*Birthweight*	*n* = 645,276	*n* = 11,078
< 1500 g	85,898 (13.3)	9,906 (89.6)
*5*^*th*^* minute Apgar score*	*n* = 504,569	*n* = 8,775
≥ 7	482,122 (95.6)	5,211 (59.4)

*n* number, *n* absolute frequency, *RDS* Respiratory distress syndrome, *C-section* Cesarean section

For the spatial analysis, 37 preterm live births were excluded due to unknown municipality code. Considering all RDS-associated neonatal deaths, delivery and death occurred in the same maternal residence municipality for 7,890 (71.2%) preterm live births. In 2,763 (24.9%) RDS-associated neonatal deaths, delivery and death occurred in the same municipality, but these were different from maternal residence municipality. In the other 425 (3.9%) RDS-associated neonatal deaths, there were different combinations of municipalities of maternal residency, infant’s birth, and death.

**Fig. 3:**
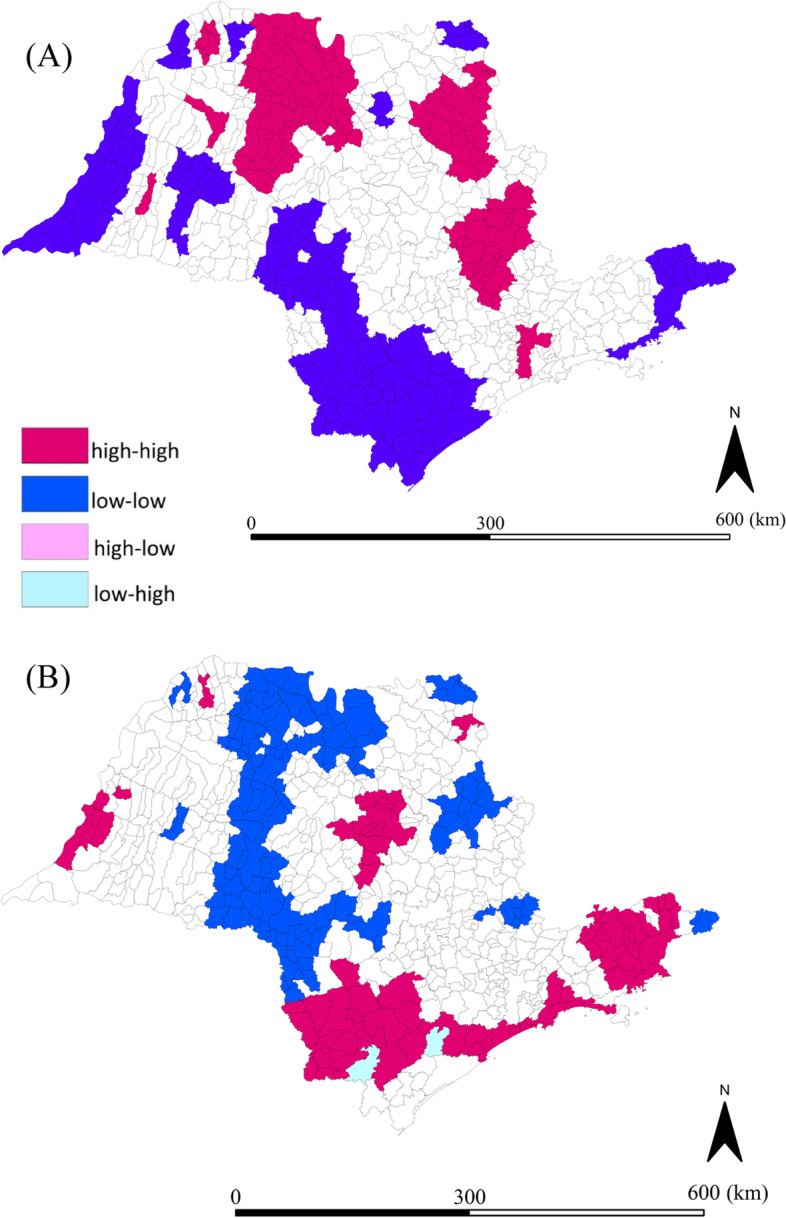
Distribution of preterm live birth and RDS-associated neonatal mortality rates. Data from 645 municipalities after smoothing using local Bayes estimate. **A** Lisa Cluster Map of preterm live births rates; **B** Lisa Cluster Map of RDS-associated neonatal mortality rates

For all 645 São Paulo state municipalities, the mean preterm birth rate during 12-year study period was 86.9 per thousand live births, with crude rates varying from 41.1 to 149.8 per thousand live births, and from 64.1 to 132.4 for spatial moving average. The Global Moran indicator suggested a non-random distribution (I = 0.44; *p* = 0.001) of preterm live birth rates across the State. After smoothing, the mean preterm live births rate for all 645 São Paulo state municipalities was 88.8 per thousand live births (range: 57.0 to 131.0) (Additional file 1), and the Global Moran indicator confirmed the presence of clusters of preterm live births (I = 0.78; *p* = 0.001). Lisa Cluster Map showed clusters of high preterm live births rates in Northwest and Midwest regions of the state and in São Paulo City Metropolitan region (Fig. 3A).

Across the 645 municipalities studied, the mean RDS-associated neonatal mortality rate was 16.2 per thousand preterm live births. The crude rate was zero in 149 (23.1%) municipalities of São Paulo State. In the other 496 municipalities, it varied from 1.9 to 95.2 per thousand preterm live births. Spatial moving average varied from 2.3 to 57.0. Evidence of non-random distribution of RDS-associated neonatal death rates was confirmed by the Global Moran indicator (I = 0.28; *p* = 0.002). After smoothing, the mean RDS-associated neonatal mortality rate for the 645 São Paulo state municipalities was 16.5 per thousand preterm live births (range 3.8 to 62.7) (Additional file 2). The Global Moran indicator confirmed the presence of clusters of RDS-associated neonatal mortality (I = 0.73; *p* = 0.001) and the Lisa Cluster Map found clusters of high RDS-associated neonatal mortality rate in Northeast, Central Coast, Southeast, and West regions of São Paulo State (Fig. 3B).

**Fig. 4:**
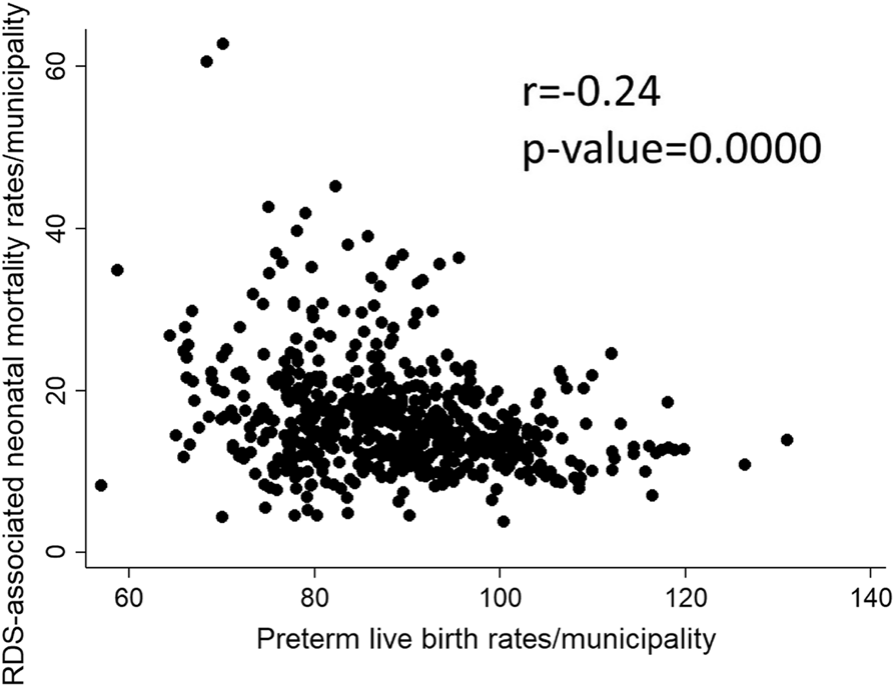
Spearman rank correlation of preterm live birth rates versus RDS-associated neonatal mortality rates

Spearman rank correlation (r) test applied for all municipalities’ rates showed a significant negative correlation between preterm live births rates and RDS-associated neonatal mortality rates (*r* = -0.24; *p* = 0.0000) (Fig. 4).

**Fig. 5:**
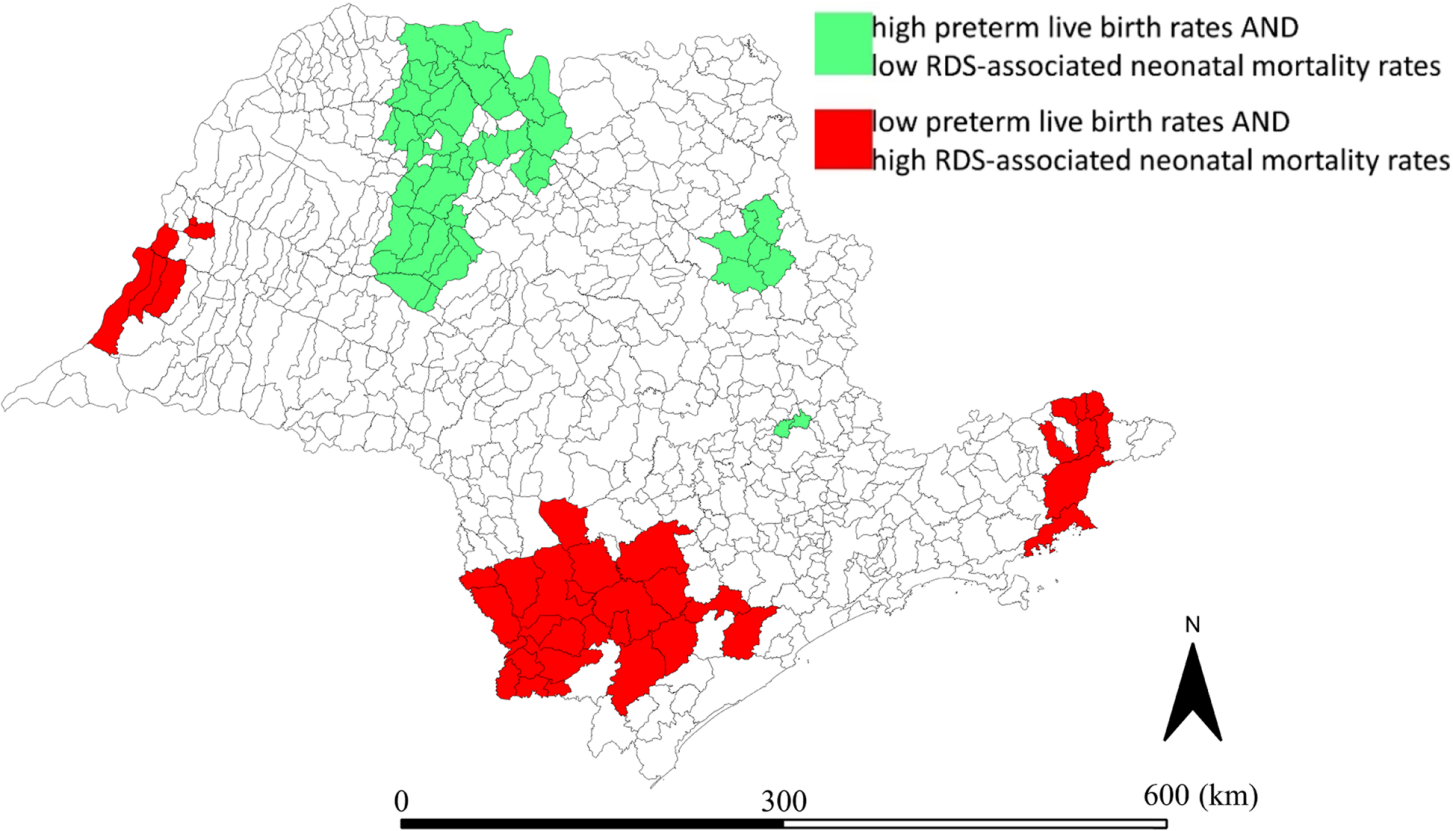
Map with asymmetries of clusters between preterm live birth rates and RDS-associated neonatal mortality rates

The overlapping of clusters identified in specific LISA cluster maps after smoothing for preterm live births rates and RDS-associated neonatal mortality rates allowed the identification of 3 municipalities with high rates for both; 34 with low rates for both; 38 with low preterm live birth rate and high RDS-associated neonatal mortality rate; and 55 with high preterm live birth rate and low RDS-associated neonatal mortality rate (Fig. 5).

## Discussion

This population-based study with a large database evaluated the spatial pattern of preterm live births rate and RDS-associated neonatal mortality rate within a highly relevant sub-national area, i.e., São Paulo State, Brazil, during a 12-year period. Across the state, a significant negative correlation between preterm live birth rate and RDS-associated neonatal mortality rate was identified, with areas of high preterm live births and low RDS-associated neonatal mortality rates, and areas with high RDS-associated neonatal mortality rate despite low preterm live births rate.

Given the strong association between prematurity and RDS, and the impact of RDS-associated neonatal mortality among preterm live births in LMIC [6, 28], it was expected an overlap of areas with high preterm live births and high RDS-associated neonatal deaths and areas with low preterm live births and low RDS-associated mortality deaths. The asymmetries shown in our study between preterm live births and RDS-associated neonatal deaths raise questions about the role of factors such as neonatal intensive care volume of admissions, neonatal team expertise, and integrated and effective regionalization policies for the care of preterm neonates in RDS-associated mortality [29–33].

Higher preterm live birth rates and lower RDS-associated neonatal mortality rates were found in the Northwest municipalities of São Paulo State, which is a region with good health indicators [34]. Northwest region has large referral centers for neonatal care and specialized teaching hospitals, with availability of neonatal intensive care beds and professional expertise [35]. Although the present study did not evaluate preterm hospitalizations, it has already been demonstrated that almost 95% of preterm live births < 34 weeks of gestational age require specialized neonatal intensive care and admission rates increase in lower gestational ages [36]. Thus, it should be expected that areas with higher preterm live births have higher hospitalization rates; the opposite also being true. In addition, higher volume of hospital admissions of high-risk preterm newborn infants has previously been associated with positive impacts in outcomes of preterm live births [37, 38]. Volume of specialized care impacts on expertise of professional teams, and efficiency of management strategies [34, 35], reducing neonatal respiratory failure and RDS-specific mortality [6, 35].

Areas with high rates of RDS-associated neonatal mortality and low preterm live birth rates found in the Northeast, Southeast, and West regions of São Paulo State could result from an inadequate regionalization process. Financial inequities, healthcare fragmentation, disproportion in hospital beds availability [29, 39], and inefficient care showed in some municipalities could impair the regionalization process [40] and the perinatal outcomes [30].

A lack of integration among healthcare levels, with deficiencies in the referral system and in regional guideline implementations, may result in maternal decisions to have the delivery in specific hospitals based on their location or on local technology availability [29, 37, 41]. In Brazil, a study done from 2011–2014 showed that wandering of women in labor in order to find a place to give birth increased the chance of neonatal death in 4.89 times [42]. Wandering was related to the lack of perinatal healthcare organization, with the absence of a pre-assignment hospital to give birth during prenatal care, and with the lack of place or lack of resources for high-risk births [42, 43]. Birth in places without the appropriate technology to promote initial care accounts for 50% of deaths in very low birth weight preterm infants [42], with a negative impact on cause-specific mortality [41, 44].

Areas with high RDS-associated neonatal mortality are also concentrated in regions characterized by the highest rates of social vulnerability and lower socioeconomic status, with younger mothers, lower maternal schooling, and lower prenatal care visits [17, 45]. All these factors are probably associated with higher neonatal mortality rates due to factors that include a poorer access to appropriate newborn care interventions [46] and to RDS good practices management strategies, contributing to RDS-associated neonatal deaths [6].

Despite the evidence that RDS prevention (prevention of prematurity and use of antenatal corticosteroids) and treatment (surfactant, appropriate oxygen and ventilatory strategies and quality of intensive care) combined have the potential to prevent 42% of RDS-associated deaths in low-income countries [5, 6, 47, 48], these practices vary among Brazilian neonatal centers. Data from Brazilian Network on Neonatal Research, with 20 public university hospitals across the country, show that antenatal administration of steroids ranged from 59 to 95% in 2015 between centers and surfactant was given to around 55% of the 1,486 infants evaluated [49]. The inconsistency of RDS management practices could contribute to the pattern of the RDS-associated neonatal deaths in São Paulo State.

The present study has some limitations. The database used in this analysis is provided by SEADE Foundation after linkage and anonymization, a process that has a manual component and is time consuming. Therefore, the most recent year available to the study was 2015. This populational database does not allow the analysis of socioeconomic and health indicators associated with preterm live births rate and RDS-associated mortality rate. The contribution of RDS to the death process of preterm infants with RDS-associated deaths notified in the death certificates could not be evaluated. Furthermore, spatial analysis was based on the smallest area available for each dataset within the state (i.e., municipality) and that may have masked spatial inequalities that would be captured in smaller units regarding social and health inequities for preterm live births and RDS-associated neonatal mortality. Although valuable, this approach certainly does not consider intra-area variability and it is influenced by areas with rare events, which could lead to extreme high or low rates [26]. To overcome this limitation, the 12-year period events were aggregated, and a spatial smoothing Bayesian approach was used.

Despite these limitations, this study is one of the first to provide a picture of RDS-associated neonatal deaths distribution in a middle-income country, offering a better understanding of perinatal care in São Paulo State. The results may be used to improve the design of public health strategies, focused on access and quality of antenatal care and maternal referral to centers with qualified professionals and resources for assisting preterm infants. Therefore, this population-based study adds information to map strengths and frailties of neonatal care in the richest sate of Brazil and information is key to planning ways to achieve the Sustained Developmental Goals until 2035 [50]. The analytical approach used in this study may be replicated and help to subsidize discussions on global inequities in neonatal health care.

## Conclusions

The present study identified across São Paulo State, Brazil, clusters of preterm live births and RDS-associated neonatal deaths, with a negative correlation between both rates, suggesting asynchrony in perinatal healthcare. The occurrence of clusters with high preterm live births and low RDS-associated neonatal deaths may be interpreted as referral areas for perinatal healthcare. Clusters of low preterm live births and high RDS-associated neonatal deaths suggest the need for public health attention to improve perinatal maternal and neonatal care.

## Supplementary Information

**Additional file 1.** Distribution of preterm live births rates in São Paulo State, Brazil (2004-2015).

**Additional file 2.** Distribution of RDS-associated neonatal mortality crude rates in São Paulo State, Brazil (2004-2015).

## Data Availability

The datasets analyzed during the current study are available from the corresponding author on reasonable request.

## Abbreviations

FAPESP: Fundação de Amparo à Pesquisa do Estado de São Paulo
SEADE: Fundação Sistema Estadual de Análise de Dados
IBGE: Instituto Brasileiro de Geografia e Estatística
ICD: International Classification of Diseases
LISA: Local Indicators of Spatial Association
LMIC: Low- and middle-income countries
RDS: Respiratory distress syndrome
GA: Gestational age
BW: Birthweight
