# Supplementary material for: Clusters of preterm live births and respiratory distress syndrome-associated neonatal deaths: spatial distribution and cooccurrence patterns
Source: BMC Public Health. 2022 Jun 20;22:1226. doi: 10.1186/s12889-022-13629-4 (PMC9210662; doi:10.1186/s12889-022-13629-4)
Supplement: Supplementary file 2 — Additional file 2. Distribution of RDS-associated neonatal mortality crude rates in São Paulo State, Brazil (2004-2015). [file 12889_2022_13629_MOESM2_ESM.docx]

**Additional file 2.** Distribution of RDS-associated neonatal mortality crude rates in São Paulo State, Brazil (2004-2015).


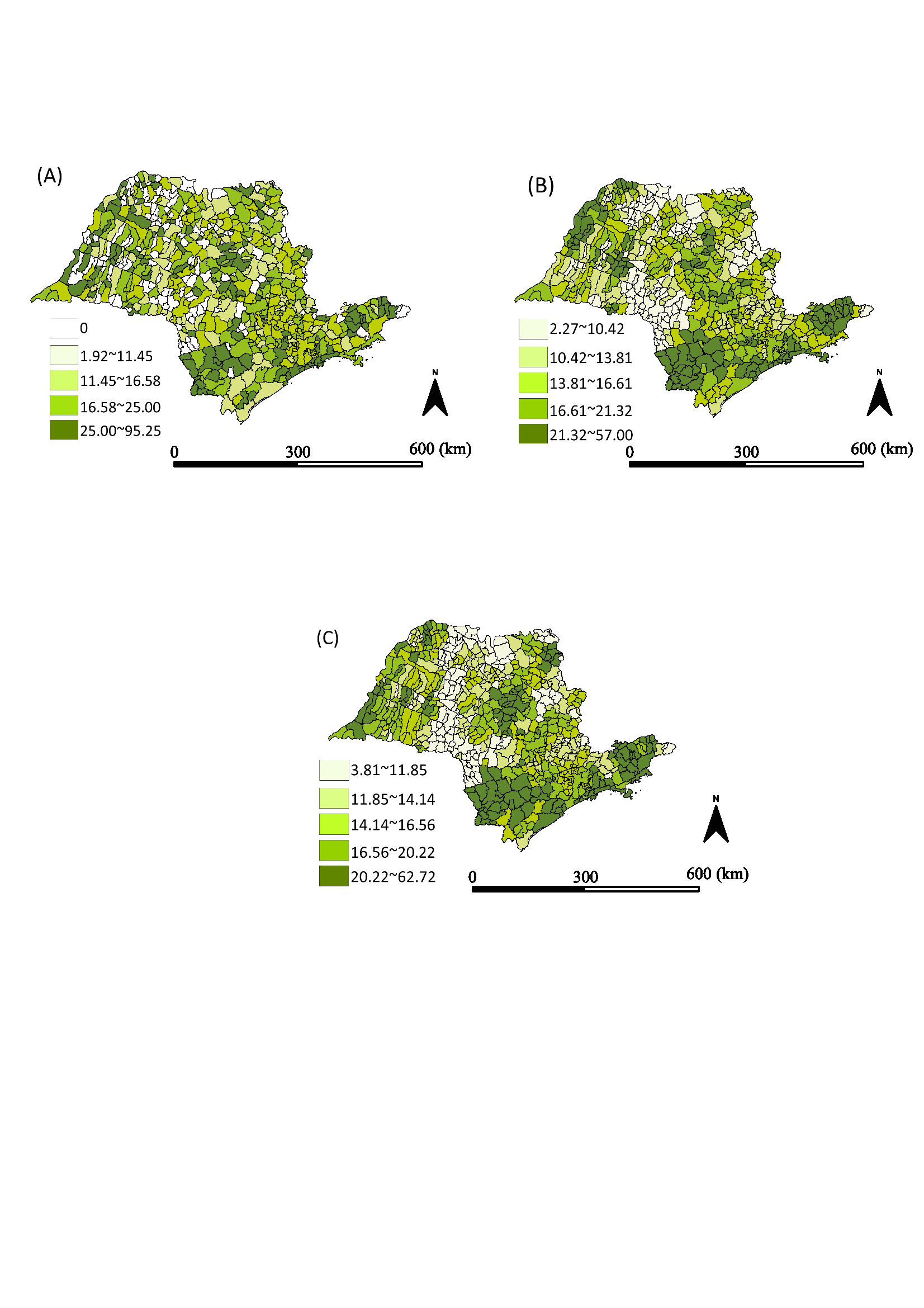


RDS-associated neonatal mortality rates distributed using quintiles. (A) Crude rates; (B) Spatial moving average; (C) Rates after smoothing.
